# 

**Published:** 1876-07

**Authors:** 


### Table of Contents.

### ORIGINAL COMMUNICATIONS.

A Plea for Principles and Conservatism in the Treatment of Diseases Peculiar to Females, Embracing the Report of a Case of Superinduced Pseudo-cyesis, with Results. *By Wm Abram Love, M.D., Atlanta, Ga.* .... 193

New Method of Performing Circumcision for Phimosis. *By Jno. Thad. Johnson, M.D., Atlanta, Ga.* .... 210

Dermoid Cyst of the Ovary. *By L. P. Rosser, M.D., Conyers, Ga.* .. 211

### ATLANTA ACADEMY OF MEDICINE.

Stone in the Bladder .... 213

Elongated Cervix Uteri in a Patient 18 years old .... 214

Artificial Dilatation of the Os Uteri as a means of hastening Natural Labor .... 217

Treatment of Malignant Dysentery .... 226

Specimen of Calculus of the Phosphatic Variety .... 228

Specimen of Enucleated Eye-ball .... 228

Abscess in the Orbit in a Child Five Months Old .... 230

Tertian Intermittent Fever Cured by Sulphate of Cinchonidia .... 231

Therapeutic Action of Muriate of Ammonia on Fibroid Tumors .... 231

### OUR EXCHANGES.

"Calamities of Surgery" .... 235

### BIBLIOGRAPHICAL.

Cyclopœdia of the Practice of Medicine .... 240

Text-Book of Human Physiology .... 241

### EDITORIAL.

Editorial Note .... 241

Proceedings of the Convention of American Medical Colleges .... 243

Twenty-Seventh Annual Meeting American Medical Association .... 249

